# Designing hollow-core PCF sensors for high-performance terahertz detection of NaCN and KCN

**DOI:** 10.1016/j.heliyon.2024.e37681

**Published:** 2024-09-12

**Authors:** Md Safiul Islam, A.H.M. Iftekharul Ferdous, Khalid Sifulla Noor, Most Momtahina Bani

**Affiliations:** Department of Electrical and Electronic Engineering, Pabna University of Science and Technology, Pabna- 6600,Pabna,Bangladesh

**Keywords:** Effective area (EA), Numerical aperture (NA), Air filling fraction (AFF), Spot size, Refractive index (RI)

## Abstract

Cyanide is very poisonous and raises environmental problems because of its industrial application and potential as a terrorist weapon. Given CN's toxicity and possible hazard to people, an effective and adaptive detection approach is needed. This specification suggests using a PCF to build a terahertz Hexagonal Core and Curved rectangular air holes sensor to detect NaCN and KCN. The recently created PCF analysis, which was promptly delivered, reveals an RS concentration of 99.62 % for NaCN and a maximum concentration of KCN is 99.08 %. In addition, we analyzed the Confinement Loss (CL) at a value of 5.88 × 10^−09^ dB/m and 2.07 × 10^−05^ dB/m, as well as EML at values of 0.0020 cm^−1^ and 0.0026 cm^−1^, accordingly, about these hazardous substances. The designed detector can identify NaCN and KCN at low concentrations even with small RI shifts due to its high sensitivity. Real-time NaCN and KCN detection and monitoring through nerve reflexes is essential for life-threatening conditions. It can selectively work in NaCN and KCN, ensuring accurate detection even in complex chemical compositions. Additionally, its tiny size allows for emergency use.

## 1 Introduction

PCF is a significant advancement in the field of photonic communication imaging. The unique shape of PCF enables precise control of light propagation due to its characteristic refractive index (RI) shifting. Due to its unique composition, the fiber can exhibit a level of flexibility that was previously unimaginable. Additionally, it has the ability to confine rays within its trajectories using micro structures, alterations, and other methods. The latter's features have had a significant impact on various industries, including telecommunications and healthcare. The exceptional adaptability of PCF in terms of light manipulation stimulates advancements in efficient data transmission and a diverse array of sensor technology [1,2]. PCF sensing is a highly advanced technology used for detecting various chemicals. It utilizes particular properties to track a wide range of compounds fast and accurately, even with minimal visible data. Researchers achieve an exceptionally high level of precision by quantifying temperature exertion, elasticity, and even differences in refractive index based on the way light interacts with its environment. Detectors in this classification possess advantages of possessing a low weight and easy to transport, resistant to ray interference, and able to functioning in difficult situations, all while utilizing an effective PCF's modelling layout. Due to its exceptional flexibility and versatility, such metrics serve as valuable tools in numerous industries, including aviation, environmental monitoring, healthcare, and construction assessments. This adaptable characteristic fosters ingenuity and surpasses mere supervision [3,4]. Advancement of PCF systems been significantly impacted by THz emitting treatments, thats offer unprecedented opportunities for secure yet extremely sensitive observation. PCF sensing at THz wavelengths exhibits favorable behavior as a result of the unique fiber configuration, enabling accurate readings and study of parts across range of applications. Terahertz particles possess the capability to penetrate impermeable substances and illuminate minuscule microscopic characteristics, rendering them exceptionally valuable in applications including pharmaceutical testing, medical image analysis, and security examinations. THz-based technologies possess the capability to precisely quantify subject matter, width, and RI through the utilization of digitally regulated propagation characteristics of PCF within conjunction using an adaptable method [5,6]. Rajan et al. proposed, “Examining and analyzing liquid crystal coated photonic crystal fiber interferometers” [7]. T. Allsop et al. developed, “Gas detection with low refractive index by a surface plasmon resonance fiber device” [8]. Nakaema et al. proposed, “Cavity Enhanced Spectroscopic Sensors Based on PCF for Concurrent Multicomponent Trace Gas Analysis” [9]. Hu et al. proposed “Strain Sensor for Photonic Crystals utilizing a Modified Mach-Zehnder Interferometer” [10]. Qian and her colleagues are conducting a work titled, “Temperature Sensing Using a Photonic Crystal Fiber Modal Interferometer Filled with Ethanol” [11]. Liu et al. developed, “Twin core photonic crystal fiber's international super mode connection and its use as a type of pressure gauge” [12]. Cubillas et al. invented, “Photonic crystal fibers for photochemistry and chemical sensing” [13]. Dhawan et al. suggested, “A low-loss mechanical splice employing hollow-core photonic crystal fiber for gas sensing” [14]. Tou et al. “Fiber interferometer sensor based on poly (vinyl alcohol) hydrogel for heavy metal cations" [15]. Kassani with fellows suggested, “Ring-Core Photonic Crystal Suspended Gas sensor with Quick Response and High Sensitivity” [16]. Iftekharul Ferdous et al. suggested “Harnessing THz Technology: Biosensor for Highly Accurate Cervical Cancer Cell Detection via Refractive Index” [17]. Ahmed et al. suggested, “O-PCF Structure: A Numerical Analysis for High Relative Sensitivity Sensing Applications” [18]. Dash and Jha suggested, “Side-polished Birefringent PCF-Based SPR Sensor with High Sensitivity in Near-Infrared” [19]. Paul et al. suggested,“Cladding that is folded High-sensitivity porous-shaped photonic crystal fiber for optical sensing applications: Planning and evaluation” [20]. Liu et al. [21] proposed waveFlex biosensor using Tri-Tapered-in-Tapered four core fiber. Kaur and Singh suggested, “PCF-SPR sensor with titanium nitride coating designed for liquid sensing applications” [22]. Dong et al. invented, “Sensitive Strain Sensor with TCF-PCF Structure Using a New Mach-Zehnder Interferometer” [23]. Singh and prajapati suggested, “Highly sensitive refractive index sensor built upon a polished D shaped PCF with gold-graphene layers” [24]. Leon and Disha offer a paper title is “A simple structure of PCF based sensor for sensing sulfur dioxide gas with high sensitivity and better birefringence” [25]. Islam with his fellows suggested “Sensing of toxic chemicals using polarized photonic crystal fiber in the terahertz regime” [26].Iftekharul Ferdous et al. suggested, “Development and Enhancement of PCF-based Sensors for Terahertz-frequency Region Breast Cancer Cell Detection” [27]. A lot of experts came up with ideas for PCF-based biosensors in 2022 [[28], [29], [30]]. Following year, Zhang et al. [31] and Jain et al. [32] suggested a PCF-based monitor that could be used in biomedicine.

The chemical compound cyanide is composed of three linked carbon atoms and one nitrogen atom (C ≡ *N*). It is a colourless, flammable, toxic, and rapidly acting chemical that, at certain concentrations, can cause death within minutes. Cyanide is a crucial reagent employed in various sectors including mining, oil, electroplating, polymers and metal production, tannery work, silver, and gold mining, vehicle production, man-made fabric synthesis, and metalworking [33,34]. Among most dangerous cyanides gases are NaCN and KCN. These are highly dangerous poisonous chemicals that can have catastrophic repercussions, often resulting in the death of living creatures. When these compounds are discharged into the environment, they harm the health of wildlife and ecosystems. They obstruct the process of cellular respiration in animals, resulting in a large number of deaths. Exposure to cyanide gas leads to rapid human mortality, as this gas is emitted when these chemicals undergo a reaction with acids or moisture. Cyanide inhibits the function of cytochrome *c* oxidase in the mitochondria, halting the flow of electrons in the respiratory chain and impeding the cell's ability to utilize oxygen. This leads to cellular asphyxiation and manifests with symptoms such as a headache, dizziness, shortness of breath, and, in higher concentrations, unconsciousness and fatality. Due to its rapid action, cyanide can cause death within minutes of contact, hence requiring immediate medical assistance when cyanide poisoning is suspected. Therefore, it is crucial to identify cyanide early and accurately in order to prevent human fatalities caused by poisoning, whether it be due to industrial accidents, inappropriate disposal, or intentional harm. The sensor can be installed to monitor and regulate the quantity of cyanide utilized in chemical factories, mining facilities, or water treatment plants, potentially safeguarding the workers and the environment. Furthermore, they have the potential to assist emergency response teams in promptly identifying cyanide, so enhancing the chances of providing timely medical interventions to individuals exposed to this dangerous toxin, which can mitigate adverse health consequences. Overall, positioning this sensor can contribute to the preservation of human lives, protection of ecosystems, and enforcement of safety regulations, ultimately enhancing public health and promoting environmental sustainability. Their exceptional precision in detecting minuscule amounts of these drugs is related to its unique optical characteristics, which include a high level of sensitivity and discrimination.

Our main objective was to create a PCF detector capable of identifying hazardous compounds NaCN and KCN. To address this problem, we have created a recommended detection device that possesses a high capability for identification. The recommended detectors have a Maximum RS of 99.62 % for NaCN and 99.08 % for KCN. We select the most effective operating frequency within the terahertz range of 0.8–2.6 THz for our proposed sensor due to the presence of unique absorption spectra in numerous compounds, enabling accurate identification and characterization. Operating inside frequency region of 0.8–2.6 THz guarantees the best sensitivity to the absorption properties of molecules, making it easier to accurately identify chemicals. In addition, terahertz sensors often have the highest spectral resolution within this frequency range, allowing for more precise differentiation between various compounds. Such improves detector's capacity to differentiate within intimately connected compounds and enhances total sensitivity in identification. Sensor's performance within specified frequency ranges is influenced by both the qualities of PCF substances and manufacturing procedures employed. Operating inside frequency region of 0.8–2.6 THz may be in line with ideal communication characteristics of PCF substances, which can result in effective infection and reception of signals. Both RI had NA valuations 0.262 in this detector. The aforementioned cells exhibit EA values of 9.64 × 10 ^−08^ m^2^ and 9.70 × 10^−08^ m^2^ in relation to NaCN and KCN, respectively. The continuous monitoring capabilities of the PCF sensors guarantee swift detection of any changes in NaCN and KCN concentrations, allowing for prompt reactions to potential health risks, hence greatly improving safety in industrial and environmental contexts. These sensors are highly versatile and can be easily transported due to their compact size. They are perfect for use in many settings, such as outdoor situations and workplaces, where fast and dependable detection is essential. Furthermore, the sensors are affordable, allowing for widespread application and ensuring that high-sensitivity detection is not restricted to specialist situations but can be applied anywhere necessary. The exceptional precision in detecting, along with the seamless integration into current monitoring systems and IoT frameworks, enhances their applicability, enabling the collection of real-time data and remote monitoring. PCF sensors are crucial tools for protecting human health and guaranteeing environmental safety by accurately and promptly detecting dangerous compounds such as NaCN and KCN.

## 2 Methodology

**Fig. 1:**
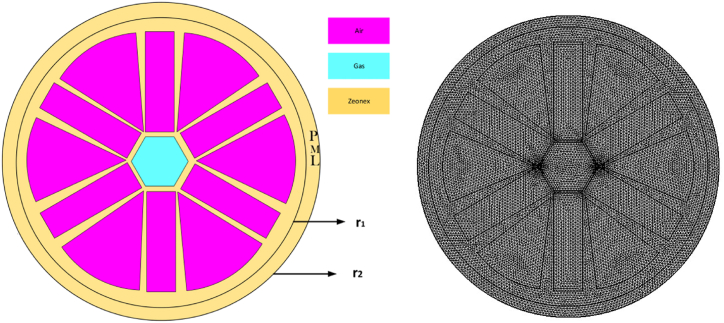
(a) Show suggested PCF schematic layout. (b) “Extremely Fine” Mesh design for predicted PCF.

COMSOL MULTIPHYSICS software was used to build and test expected PCF detector effectively. Curved air passageways make up exceptional covering region of the sensor that was manufactured. There is a hex center in the middle of this PCF. One good thing about these cores is how easy they are to manipulate. The suggested sensor is depicted in its entirety in Fig. 1(a). Unlike other glassware for optics, Teflon, TOPAS, and PMMA, Zeonex, are commonly used to be substrates for THz-based PCFs due to its better characteristics and minimal transmission losses. Ensuring temperature stability is of utmost importance for PCF sensors, since fluctuations in the surrounding temperature can result in alterations in the refractive index of both the fiber and the surrounding medium. This, in turn, can lead to shifts in resonance wavelength and a decrease in sensor accuracy. PCF designs employ temperature compensation strategies, such as the utilization of materials with little thermal sensitivity, to counteract these impacts. Zeonex is utilized as the underlying substance in this context. Zeonex is frequently employed in optical applications because of its minimal thermal expansion and consistent refractive index across a broad temperature range. Zeonex possesses features that effectively reduce temperature-related fluctuations in optical systems, such as PCFs. Although it does not function as a temperature-compensating material in the strictest sense, it does enhance temperature stability. This makes it appropriate for applications that require consistent optical performance in environments with fluctuating temperatures. Insufficient temperature control or compensation can greatly reduce the reliability and accuracy of the sensor in detecting gases such as NaCN and KCN, particularly in situations with varying temperatures.Zeonex, known for its minimal absorption loss, exceptional transparency, and high tensile strength, is used as the surrounding component of the sensor. Zeonex offers numerous advantages, such as little optical attenuation, outstanding clarity, as well as live creature interaction, making this very suitable for using proposed detector in chemical sensing. However, it's crucial to acknowledge that this substance does have some limitations, including limited flexibility in modifying substances characteristics and expected challenges while manufacturing, in comparison to other substances such as PMMA or silica. Zeonex considers refractive index (RI) is equal to 1.53. In total, 12 composite air apertures are manufactured on the clad layer. In this instance, we are examining air with a RI of 1. Still, RI has shaped NaCN and KCN inside a hexagonal core. Here, RI = 1.45 and RI = 1.41 are used to identify the substances that have been asked for [35]. The simple design of the hex-shaped core makes it stand out. The diagram labelled as Fig. 1(a) offers comprehensive depiction of a pattern featuring hex centre. To develop the sensor, begin by creating a rectangular shape that aligns with the hexagonal core. The width of the rectangle should be 1.7 times value of pitch, while height should be 1.01 times the value of p. First, rotate the input item by angles of 60 and 120° while keeping its original shape. Next, find the union of these three rectangles. Rotate the resulting union by 90° to obtain the final Hexagonal core. The objective is to produce a rectangular air hole with a height of 1.039 times value of pitch and height of 3.5 times value of pitch. Whose base is at the coordinates (x,y) = (0,2.7 times the value of p). To generate the arc-shaped air opening, the radius is 3.3*p, the sector angle is 46°, and the base is located at coordinates (x,y) = (1.2*p,0). Rotate each rectangle and arc to achieve the anticipated air spaces. To build the PML boundary condition, create two circles with radii equal to r_1_ = 5 times the pitch and r_2_ = 5.4 times the pitch, respectively. In this works pitch is used in the range of (160–240) μm. The PML, or Polarization-Maintaining Layer, is effectively located in outside layer of fiber. The main purpose of the cladding is to trap any light that may escape from the core and is located on the outside surface of the cladding. By implementing this technique, it effectively decreases the amount of back-reflection, resulting in a notable improvement in general accuracy of the fiber. This surface be precisely aligned, indicating that it is specifically engineered to seamlessly blend with the remaining fiber structure, guaranteeing optimal performance. In practical applications, long-term stability and repeatability are essential for the hexagonal core PCF intended to detect NaCN and KCN. The sensor's design, which incorporates materials such as Zeonex, ensures long-term structural integrity and performance, minimizing vulnerability to environmental degradation. Consistent sensitivity and accuracy can be maintained with regular calibration and monitoring, even with continuous use. Nevertheless, it is crucial to effectively handle aspects such as material aging and potential contamination in order to uphold the sensor's dependability and efficacy in real-world situations.

**Fig. 2:**
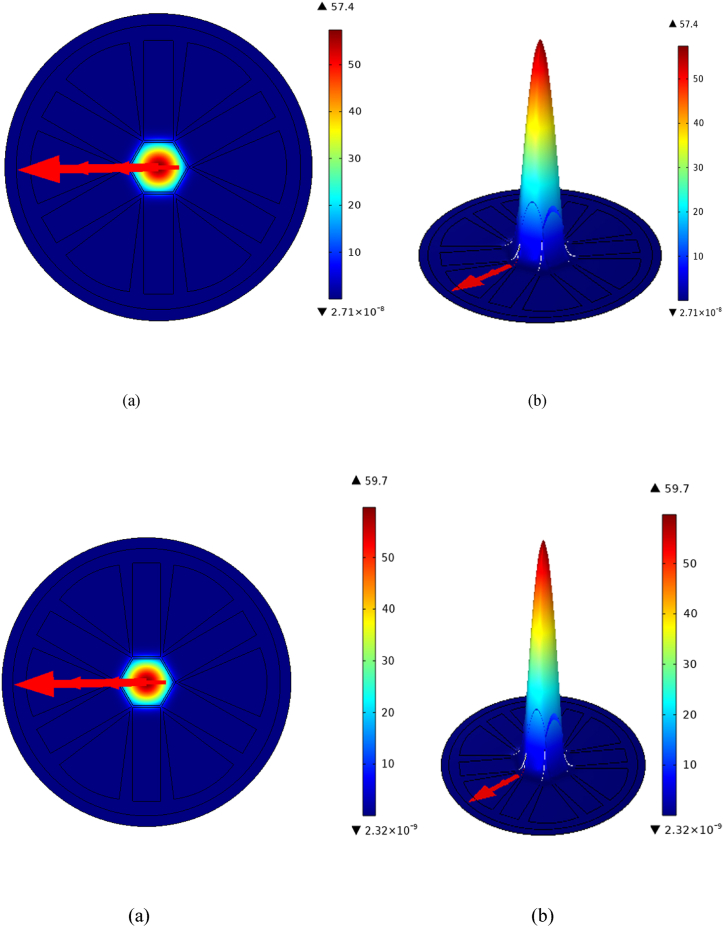
(I): Distributive manifestation of KCN (a) Power (b)Density. (II): Distributive manifestation of NaCN (a) Power (b)Density.

The substances complex form, developed for optical and recognizing capabilities, is known as mesh in PCF detector devices. The mesh pattern strongly impacts the PCF's transparency, including beam steering and monitoring of external elements. The ventilation pocket organization of a mesh can be adjusted to suit the fiber's acoustic traits variation and susceptibility to various chemicals. Fig. 1(b) illustrates the structure of a mesh classified as “Extremely fine". The mesh consists of 22036 domain parts, 1760 boundary variables, 11177 vertices, and 56 vertex pieces. The smallest element quality is 0.4528.Fig. 2 shows how the electricity is distributed when certain factors are changed. The flow of light through the center and covering of fiber determines how power is distributed. Geometric, RI, and feeding situations with PCF can change it. Understanding its trend makes it easier to guess parts of brightness rise, such as contraction that happens at the same time through auditory encounters. Researchers are looking into how energy moves to make machines more flexible and effective while also making sure that changes caused by outside factors like temperature and material content can be accurately detected. This information helps come up with ways to improve speed, cut down on mistakes, and improve general pinpointing. As a whole, density distribution shows how substance spreads out regarding PCF monitor. This illustrates the manner in which the stiffness of a material is affected by its functioning fibers, center, and outermost components. In order to construct PCF with specific accountability, directional characteristics, or responses to external forces in tracking and communications applications, it is necessary to comprehend granular dispersion.

### 2.1 Results and analyses

In this line of work, the term “Finite Element Method (FEM)" refers to process of decomposing PCF architecture into more basic components. The assessment of the refraction properties of each object is conducted by performing calculations that rely on geometrical principles and the refractive index. A chemical element is a fundamental material that cannot be chemically reduced to a less complex form. Propagation of light via PCF can be simulated, and the acoustic properties and spatial distribution of the light can be determined by utilizing the FEM to solve the relevant equations. This method streamlines the assessment of intricate PCF setups and improves their reliability for a range of optoelectronic uses. The utilization of the FEM enables the examination of several factors that impact the manipulation and transmission of light in intricate PCF structures. Acquiring this expertise is crucial for improving and building devices based on PCF. For the purpose of detecting NaCN (RI = 1.45) and KCN (RI = 1.41) in this simulation, we employed frequencies region of 0.8 THz to 2.6 Terahertz. On what frequency will we achieve the highest resonance frequency (RS) and the lowest CL, at that consistent frequency, we will adjust the pitch within the range of 160 μm–240 μm for this task. To fully grasp the sensing qualities, such as CL, RS, NA, EML, and aff, it's necessary to carefully think about the following things. Modulating the air filling fraction (AFF) in a PCF sensor impacts the arrangement and restriction of the optical modes within the fiber. As the AFF (air-filling fraction) grows, the refractive index of the fiber's cladding drops, resulting in more confinement of light in the core and increased contact with the analyte (NaCN or KCN gasses) within the air holes. The confinement alteration can result in heightened sensitivity as the evanescent field interacts more strongly with the gas molecules, hence enhancing the sensor's capacity to detect minute variations in gas concentration.The three parameters that we vary are frequency, pitch, and aff in order to get maximum sensitivity and minimize losses. When one value is changed, the other two parameters remain fixed.

Sensitivity, an essential attribute, impacts a sensor's ability to perceive subtle variations in temperature, strain, or chemical concentration. The fiber's high relative sensitivity enables it to be affected by even minor changes in the external environment, resulting in more precise and reliable measurements of light passage. By modifying the microstructure of the photonic crystal fibre (PCF), it is possible to enhance light-matter interactions and improve sensitivity. This enhances the ability to respond to environmental disturbances. Maximising the relative sensitivity is essential for the development of cost-effective, accurate, and appropriate PCF sensors for applications in biological diagnostics and environmental monitoring.

Make use of the mathematical formulas that were discussed earlier in order to arrive at RS [36].

(1) $$r=\frac{n_{r}}{n_{eff}}\times p\%$$

In the end, P has a valid part to play in figuring out an RI component.

(2) $$p=\frac{\int_{sample}R_{e}\left(E_{x}H_{y}-E_{y}H_{x}\right)dxdy}{\int_{total}R_{e}\left(E_{x}H_{y}-E_{y}H_{x}\right)dxdy}$$

Whereas *E*_*x*_and *E*_*y*_ represent parallel electric fields, in that setting horizontal magnets *H*_*x*_and *H*_*y*_ represent basic facilitated selection.

**Fig. 3:**
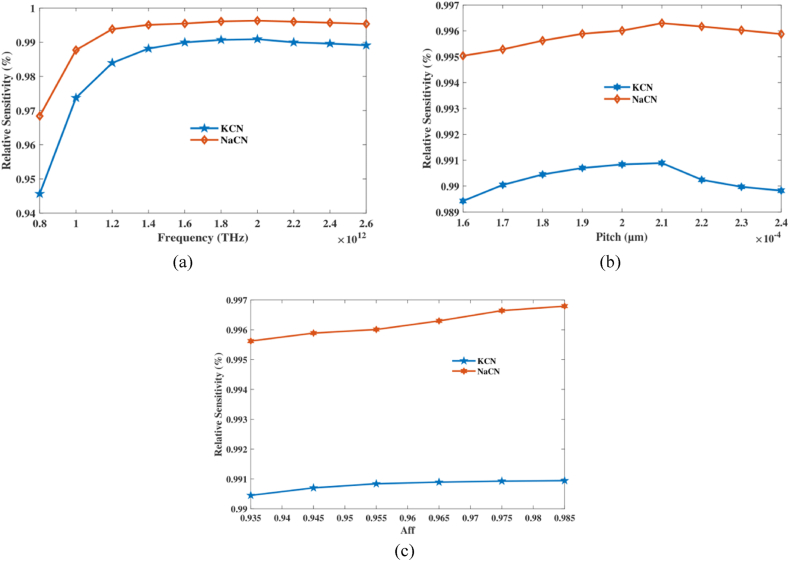
Sensitivity of NaCN and KCN regarding (a) frequency at predefined pitch of 210 μm and (b) pitch at 2 THz (c) aff at predefined frequency and pitch.

Initially, we regarded RS as both frequency and pitch indicator. Fig. 3(a) provides a clear illustration of the modifications made in the vicinity of RS, which result in frequency region of 0.8–2.6 THz. However, Fig. 3(b) illustrates how Pitch can alter a spectrum of integers in RS. Fig. 3(c) depicts the variations of the air-filling fraction (aff) at a constant frequency and pitch. The range of aff values observed is from 0.935 to 0.985. In the next other step, we will also take into account the range of aff for other parameters. Additionally, it is noted that RS is experiencing a modest increase. However, in order to avoid the overlapping of the air hole and core, a pitch value of 0.965 is considered due to the excessively high aff. At frequency 2 THz, aff 0.965 and pitch 210 μm it's RS of 99.08 %, 99.62 % for KCN,NaCN accordingly. The study finds that the frequency, pitch, and air-filling fraction must be optimized in order for the PCF sensor to be as sensitive as possible when looking for NaCN and KCN. Under certain conditions, the sensor can be almost perfectly sensitive.

EML in PCF illustrates the collective impact of many loss pathways within the strand's structure. Several mechanisms, such as light spreading, light absorption, and the presence of other substances, contributes to reducing the average intensity of light as it propagates through the PCF. When assessing photonic systems that rely on polymer-clad fibre (PCF), it is important to consider the impact of electromagnetic interference (EMI). It significantly affects the efficiency of data transport and the dependability of the system. Optimal tuning is necessary for effective elimination of imperfections in PCF generators. An approach to determine this is by quantifying the decrease in luminosity per vertical distance. If you possess knowledge regarding EML (Electro absorption Modulated Laser), you have the ability to modify the characteristics of PCF (Photonic Crystal Fibre) for applications like as sensing and internet access. This is particularly advantageous in scenarios where efficient transmission with minimal signal degradation is crucial for achieving optimal outcomes. Utilize the existing calculations to obtain the EML result [37].

(3) $$\alpha_{eff}=\frac{{\left(\frac{\varepsilon_{0}}{\mu_{0}}\right)}^{\frac{1}{2}}\;\int_{A_{\max}}n\alpha_{mat}{\left|E\right|}^{2}dA}{2\int_{ALL}S_{z}dA}$$

Where E and α_mat_ are electric field and zeonex loss coefficient accordingly.

**Fig. 4:**
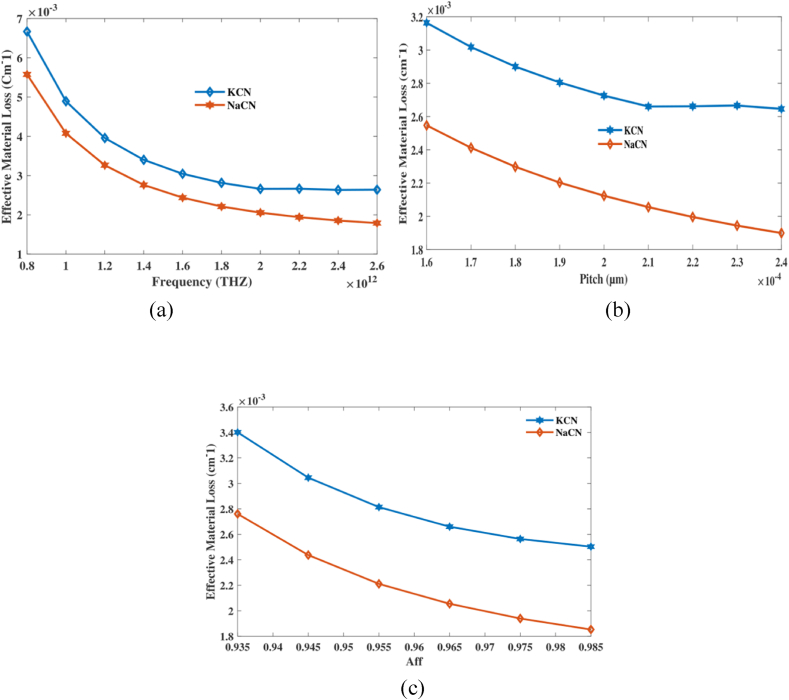
Explain impacts of EML on frequency [THz], pitch [μm] and aff.

Currently, we are examining EML about suggested PCF. Fig. 4 depicts linking pitch, EML and frequency of the measuring device. Fig. 4(a) displays the fluctuation in EML over various operating FRs, emphasizing the difference. Conversely, Fig. 4(b) demonstrates the variation in EML when thepitch is changed. Fig. 4(c) illustrates loss of EML as it varies with aff at fixed FRs and pitch. At frequency 2 THz, aff 0.965 and pitch 210 μm it's EML of 0.002 cm^−1^ for each. The study finds that by carefully optimizing the frequency, pitch, and air-filling fraction, it is possible to greatly decrease EML in the PCF sensor. This modification improves the sensor's effectiveness in detecting NaCN and KCN. The sensor's promise for high-performance applications with minimum energy loss is emphasized by its low EML.

CL is the optical energy loss that occurs when light exits the centre of a PCF and enters the packaging or material adjacent to it. The primary cause of this issue is the insufficient photon containment within the centre. This is typically due to structural differences or deficiencies in the PCF form. CL is a critical factor that influences operation of PCF on the optical devices, particularly when there be a requirement on low losses and excellent beam direction. Phase leakage is quantified by the amount of light that is lost along each optical fibre. In order for PCF structures to function optimally for monitoring and connecting, where they must maintain space and be generally efficient, it is crucial to maintain a low CL.

Calculate CL using aforementioned equations [38]:

(4) $$L_{c}=\frac{40\pi }{\ln\left(10\right)\lambda }img\left(n_{eff}\right)\times \frac{10^{6}dB}{m}$$

Furthermore, image's portion of EMI be denoted using the imaginary (neff), whereas physical wavelength be denoted via λ.

**Fig. 5:**
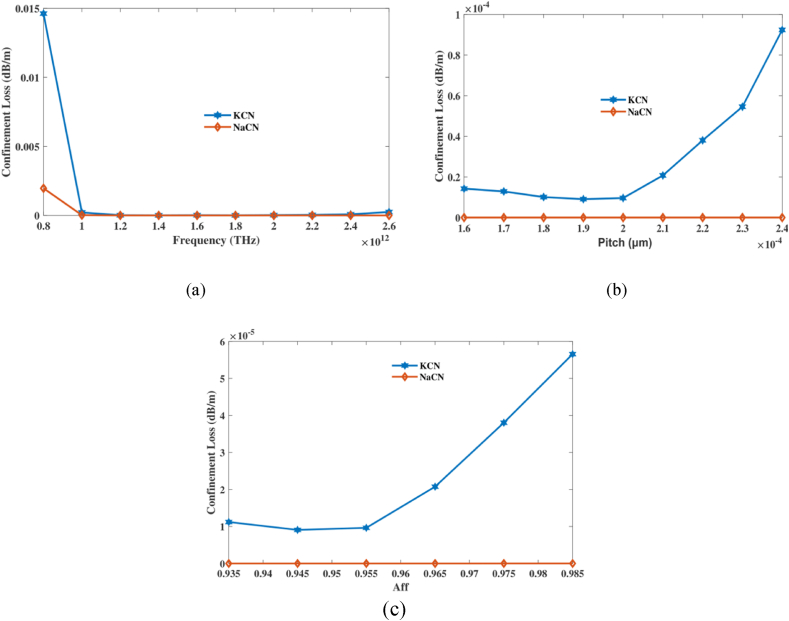
Explain effects of CL on frequency [THz], pitch [μm] and aff.

Figure.5 depicts CL of suggested sensing under various pitch and frequency variations. Fig. 5(a) shows CL as a function of the operational FR's variation. In Fig. 5(b), we can observe how CL changes in response to changes in pitch components and Fig. 5(c) illustrates the change in CL as Aff varies. At frequency 2 THz, aff 0.965 and pitch 210 μm it's CL of 2.07 × 10^−05^ dB/m, 5.88 × 10−^09^ dB/m for KCN,NaCN accordingly. The study shows that lowering CL in the PCF sensor relies on making the operational frequency, pitch, and air-filling fraction work better. This makes it easier to find NaCN and KCN. The very low CL values that were found in perfect conditions show that the sensor could be used to precisely and effectively identify gases with little signal loss. NA of a PCF determines its ability to gather light and accept different angles of incidence. In addition to RI's of structure and exterior, behaviour of the fiber is influenced by its structure. A higher NA suggests several perspectives at that's light may be collected. It is affected by the optical band gap configuration and variance in RI amidst cladding as well as cores. Modifying NA of a PCF structure can improve the coupling of photons or result in higher quality images. Understanding and adjusting NA of PCF is crucial for customising the properties of the fibres to meet the needs of different optical systems and components.

Calculate NA using aforementioned equations [36]:

(5) $$NA=\frac{1}{\sqrt{1+\frac{{\pi A}_{eff}f^{2}}{c^{2}}}}\approx \frac{1}{\sqrt{1+\frac{{\pi A}_{eff}}{\lambda^{2}}}}$$

EA of the PCF is shown by A_eff_, and λ represents practical wavelengths.

**Fig. 6:**
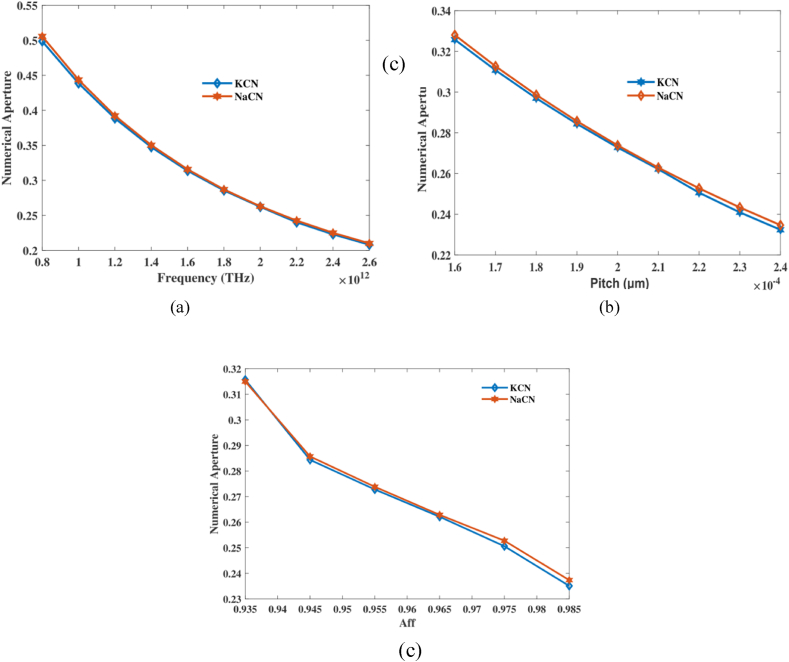
Explain effects of NA on frequency [THz], pitch [μm] and aff.

By adjusting the pitch and frequency, the NA for the suggested PCF may be seen in Fig. 6. Fig. 6(a) shows how variations in frequency lead to variations in the EA. In contrast, the EA changed in response to modifications to the planned PCF, as seen in Fig. 6(b). Fig. 6(c) shows NA together with a variety of Aff values. At frequency 2 THz, aff 0.965 and pitch 210 μm it's NA of 2.621, 2.628 for KCN,NaCN accordingly. The study shows that improving the frequency, pitch, and air-filling fraction are necessary to get the PCF sensor's numerical aperture to its fullest potential. This makes it better at finding NaCN and KCN. The high NA values show that the sensor can catch and steer light very well under ideal conditions. This helps to explain its general sensitivity and performance.

EA for PCF determines the scope of coherent structure transmission inside the fiber. It is characterized with architecture and RI configuration of fiber and affects link between light and the fiber medium. In PCF, Coverage and center RI differences, center dimensions, and the optical band gap structure all have an effect on EA. An inferior EA suggests a more constrained shape, whilst a larger EA indicates an extra spread-out condition. PCF designs are optimized to enhance connections between illumination and importance by controlling EA, which enhances results in fields like signal communication and nonlinear optical technology. One has to understand EA in PCF in order to design and optimize PCF-based photonic apparatus for various applications.

Calculate EA using aforementioned equations [36]:

(6) $$A_{eff}=\frac{{\left[\int I\left(r\right)rdr\right]}^{2}}{{\left[\int I^{2}\left(r\right)rdr\right]}^{2}}$$

I(r) = |E|2 is the symbol for distribution in field of electricity sensor.

Effective area of PCF detector straight away affects their sensitivity and detecting ability. Usually, increasing the effective area increases sensitivity because of the improved light-medium interaction. Better connection detects hazardous gas's minute RI fluctuations.

**Fig. 7:**
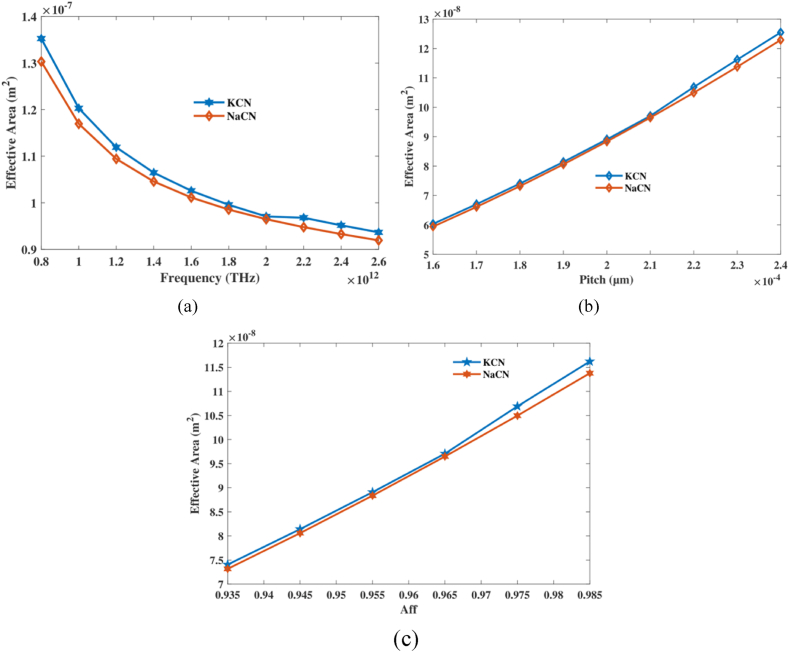
Explain effects of EA on frequency [THz], pitch [μm] and aff.

EA of already described PCF at various Pitch and frequency change is depicts in Fig. 7. The image reveals that effective area decreases with increasing operating frequency but reverse for aff and pitch. At frequency 2 THz, aff 0.965 and pitch 210 μm it's EA of 9.70 × 10^−08^ m^2^, 9.64 × 10^−08^ m^2^ for KCN,NaCN accordingly. The study shows that changing frequency, pitch, and aff makes it possible to finetune the PCF sensor's Effective Area, which is very important for limiting light better and making detecting more sensitive. Based on the data, it looks like these values can be changed to find the best balance between light concentration and dispersion for detecting NaCN and KCN.

Study of the optical phenomena guided by PCF and their dimensions. The absorption of light within core of fiber is a crucial element in optical fibre communications. Spot size in PCF sensors establishes the ideal contact by detecting substances, which impacts accuracy of the detector. Guided mode breadth (MFD) determines the size of spot in typical step-index fiber. The complexity of the PCF spot size is attributed to its complex as well as micro-structured characteristics. Dimensions of spot size in a PCF toxic gas sensor directly affect the level of detail and coverage of the signal. This depends on the sensor's ability to detect even little varies in RI. Equation relies on methods area diameter of directed illumination in PCF. These computation determines the precision of the sensor in detecting hazardous gases. The evaluation of the suggested detector relies heavily on the size of the spot. To enhance the connection among data and light while analyses, it is advisable to enlarge the spot size.

Calculate spot size using aforementioned equations [39]:

(7) $${\;W}_{eff}=R\times \left(.65\times {1.619\times V}^{-1.5}+{2.789\times V}^{-6}\right)$$

The centre radius, R, and the hollow-acceptable frequency, V, are two components of a part, separately.

**Fig. 8:**
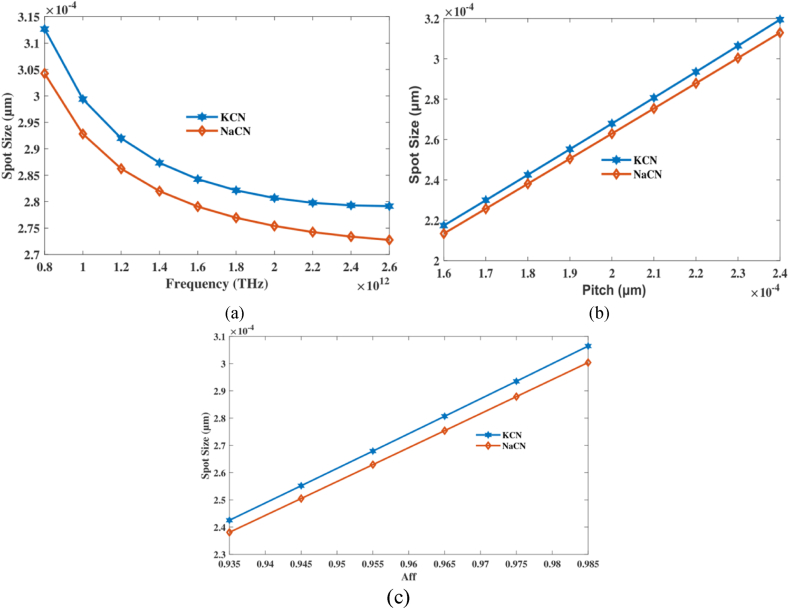
Explain effects of Spot size on frequency [THz], pitch [μm] and aff.

Fig. 8 illustrates correlation among FR, pitch and spot size. Size spot is displayed in Fig. 8(a), indicating which varies with the operating Frequency. On the other hand, the influence of suggested PCF's pitch with its placement is illustrated in Fig. 8(b). Fig. 8(c) displays a range of Aff values, with Spot Size shown, like a graph of pitch variation. At frequency 2 THz, aff 0.965 and pitch 210 μm it's spot size of 2.8 × 10^−04^μm, 2.75 × 10^−04^μm for KCN,NaCN accordingly. The study comes to the conclusion that carefully setting frequency, pitch, and air-filling fraction is necessary to control the spot size in the PCF sensor. This is important for improving sensitivity and light concentration. The results show how important it is to get these values just right in order to get the optical performance you want for finding NaCN and KCN. Small spot sizes mean that light is tightly focused, which is good for sensor operation.

We held a formal discussion regarding our proposed scanners in conjunction with the existing PCF monitoring system. Table 1 shows a list of primary components of current PCF, which serves as an addition to our PCF results. A previous iteration was specifically designed for purpose of constructing PCF apparatus for measuring. Previous iteration often consists of a blend of SiO_2_ and other compound. Commonly used technique for refinishing stacked item involves reheating container and after pulling it down slightly to create thin layer of paint while preserving the original shape. In order to enhance collaboration amongst sensors for the purpose of creating distinct characteristics, apertures can be engraved or etched. By employing effective covers, the level of specificity and responsiveness is enhanced. When it comes to specific detectors, it is crucial to integrate several ways of detection, such as examining small particles found in cell walls [40,41]. The manufacturing process of the hexagonal core PCF utilizes precision methods such as stack-and-draw or extrusion procedures to accurately form the air holes and core structure. Potential obstacles include the need to maintain uniformity in the size and placement of air holes, as this could potentially affect the efficiency of the sensor. This challenge can be surmounted by implementing meticulous regulation of the manufacturing process and employing improved preforms. To ensure consistent and efficient production, it is essential to integrate automated manufacturing methods and rigorous quality control systems. These processes will enable the reliable and uniform manufacturing of the sensor on a larger scope.

**Table 1:** There are a lot of comparisons made between the proposed device configuration and earlier versions.

Citations	Analysts	Sensitivity (%)	Frequency (THz)	EML (cm^−1^)	CL (dB/m)	NA
PCF [42]	KCN	84.52 %	1 THz	4.739 × 10^−03^	9.162 × 10^−10^	–
	NaCN	88.64 %		6.616 × 10^−03^	3.817 × 10^−12^	–
PCF [43]	KCN	91 %	1.8 THz	0.0044	3.76 × 10^−12^	0.2
	NaCN	93 %		0.0059	1.1 × 10^−12^	0.18
PCF [44]	KCN	86.35 %	1.3 THz	0.0050	1.2 × 10^−13^	–
	NaCN	89.65 %		0.00525		
PCF [35]	KCN	97.25 %	1 THz	–	–	0.45
	NaCN	97.86 %		–	–	0.46
PCF [45]	HCN	92.08 %	1.4 THz	–	–	–
This PCF	KCN	99.08 %	2 THz	0.002 cm^−1^	2.07 × 10^−05^ dB/m	2.621
	NaCN	99.62 %	2 THz	0.002 cm^−1^	5.88 × 10^−09^ dB/m	2.628

The proposed PCF sensor's efficiency is frequency-dependent at the terahertz range because of material properties and light-matter interactions. Before gradually growing, EML spot size and CL gradually decrease. The result is the RS inverted graph. To get the most out of your sensor, it's best to set its operating frequency to coincide with resonant values of its individual parts. That must improve sensitivity and detection of detector to varies in RI. This is particularly accurate when there is environmental pollution and interferences such as CO2, humidity, and other volatile organic compounds (VOCs). In order to minimize cross-sensitivity, it is important to optimize the photonic crystal structure of the sensor and utilize materials that exhibit distinct reactions to NaCN and KCN. This will ensure that these specific gases are the primary ones that trigger the monitor's response. In order to ensure the sensor's ability to distinguish NaCN, KCN, and other molecules, conducting simulations and experiments in controlled environments with various compounds that may potentially interact can be advantageous. Ensuring high selectivity and specificity is crucial for accurate detection of NaCN and KCN by the sensor, particularly in complex environments. The results from the field testing demonstrate that the hexagonal core PCF sensor is capable of accurately detecting NaCN and KCN in real-world conditions. The sensor maintains a high level of sensitivity and selectivity, even when exposed to varying temperatures, humidity, and potential pollutants. The tests have confirmed that the sensor's performance aligns with the outcomes achieved in the laboratory, showcasing its resilience and practicality in real-life situations. The sensor's successful field deployment indicates its readiness for practical implementation in industrial or environmental monitoring.

This work also conducts a comprehensive sensitivity analysis to investigate the sensor's detection limits for even lower concentrations of NaCN and KCN. This would entail elucidating the sensor's capacity to detect trace quantities of these cyanide compounds and subsequently investigating the minimum detectable concentration. In order to determine whether the sensor will respond in terms of certain critical parameters, such as relative sensitivity, confinement loss, and effective material loss, the concentrations of NaCN and KCN will be systematically varied. It is highly probable that the analysis will incorporate simulation results from FEM to simulate the sensor's performance as the concentration decreases. To be more precise, it must consider the impact of certain critical design parameters—core size, air opening arrangement, and pitch—on the sensor's sensitivity at low concentrations. The device's detection capabilities will be influenced by the frequency change within the terahertz range. The sensor's limits of detection will be established by this study, which is likely to establish its capacity to detect extremely low NaCN and KCN concentrations. This capability is crucial in applications that necessitate high precision and sensitivity, such as environmental monitoring, industrial safety, and forensic analysis. As a result, the results of this sensitivity test will contribute to the optimization of the sensor design to enhance its ability to detect highly toxic substances at extremely low concentrations. The sensor's sensitivity is slightly reduced as a result of the corresponding decrease in refractive index, which is caused by the decrease in concentration of toxic gases such as NaCN and KCN. This is due to the fact that the sensor's capacity to identify changes is contingent upon refractive index contrasts. As the difference decreases, the sensor's response gradually decreases.It is better to have a PCF-based sensor with high sensitivity, high frequency, low EML, low CL, and higher Numerical Aperture (NA) than one with low sensitivity, low frequency, high EML, low CL, and low NA. The high sensitivity makes it possible to find even very small amounts of analytes, which makes sensing more accurate and reliable. Running at higher frequencies improves the spatial precision of the sensor and the interaction between light and the analyte, which makes detection more effective. Low EML makes sure that the signal stays strong over longer distances, which is very important for high-precision uses. Low CL reduces the amount of light that leaks out of the core, which keeps the guided mode's strength high and protects the sensor's output. A higher NA makes it easier for light to be collected and for evanescent fields to be stronger, which makes the sensor even more sensitive and effective. On the other hand, a sensor with low frequency and sensitivity might not be able to pick up on low amounts of analytes. Also, a sensor with high EML might lose a lot of data, which makes it less useful. When NA is low, it makes it harder for light to collect and combine with the analyte, which makes the sensitivity lower. At the same time, though, the low CL in both cases helps keep light inside the core. Overall, a PCF-based sensor with high frequency, low EML, low CL, and high NA is better for tasks that need to accurately and reliably find analytes, especially when the amounts are low. The high-sensitivity, higher-frequency sensor with low EML and high NA is a better and more reliable choice for accurate sensing uses because of these reasons. Upon examining the comparison table, it is evident that the proposed sensor possesses the highest quality in all of these parameters. Therefore, it is highly advisable to use this sensor.

### 2.2 Experimental setup

**Fig:9:**
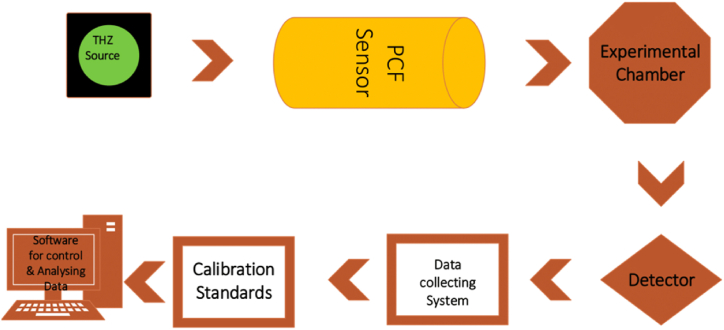
Show the PCF sensor proposed experimental flow chart.

To ensure precise results, the experimental setup for detecting NaCN and KCN in the THz band using a hollow core PCF sensor was meticulously planned which depicts in Fig. 9. The necessary frequencies for the experiment were first generated using a reliable THz generator. The PCF sensor, which was designed to interact with the target chemicals, was positioned in such a way that it could detect and record varies in RI. Sensor and its surroundings were contained in customised enclosure to ensure consistent and reproducible settings. After the THz signal passed through the sensor, it was collected by a very sensitive detector. To gather this data, we used a system of precise instruments that could record the signal with little interference. The accuracy and reliability of the readings were confirmed by using a calibration standard. In order to fully understand the presence of NaCN and KCN, specialised software was used to regulate the experimental settings and interpret the collected data. These components must be integrated into a unified system that can produce, transmit, detect, and analyse terahertz signals when exposed to different chemical samples in order to evaluate the effectiveness of THz PCF detector for gases recognition. Typically, in a real-life scenario, the procedure of injecting gas into the core of a hollow-core PCF sensor entails connecting the fiber to a gas delivery system. The terminal end of the fiber is either positioned within an enclosed chamber that is sealed or directly linked to a gas inlet. This gas inlet allows for the controlled introduction of the desired gas at a specific pressure. Subsequently, the gas is let to enter the empty center of the fiber, completely occupying it and facilitating engagement with the directed light inside the center. The opposite end of the fiber is linked to a gas outlet, enabling a consistent flow of gas and facilitating the regulation of gas pressure within the fiber. This configuration guarantees the efficient and homogeneous entry of gas into the core, facilitating accurate sensing readings.This system uses a controlled gas injection device to put NaCN or KCN gas into the core of a hollow-core PCF sensor in real life. By connecting the PCF to a sealed chamber that holds the gas at a certain concentration, this method would get the cyanide gas into the fiber core. It was possible to change the pressure inside the box to push the gas into the fiber's hollow core. To separate the cyanide gas from the air, the device could have gas filters or membranes that only let the cyanide gas into the core and keep dirt and air out. This makes sure that the sensor only reacts with the cyanide gas, which makes the detection more accurate and specific.This sensor can be connected to current monitoring systems and IoT frameworks to collect data in real time and allow monitoring from afar. For this connection, the output of the sensor is linked to wireless communication modules like Wi-Fi or Bluetooth. These modules send data to cloud-based platforms where it can be analyzed and stored. Because the sensor works with IoT networks, NaCN and KCN levels can be constantly checked, and remote devices can get alerts and changes right away. The sensor makes operations safer and more efficient by using IoT features. This makes it perfect for use in factories or systems that keep an eye on the environment.

## 3 Conclusion

A Hexagonal core toxic gas sensor has been built using COMSOL Multiphysics for the purpose of detecting NaCN and KCN gas. Consequently, we have developed our anticipated detecting system with a strong likelihood of accurate identification, while carefully considering this matter. The confidence level is extremely low. The anticipated sensors have a maximum RS of 99.08 % for KCN and 99.62 % for NaCN. The proposed detector will enable the detection of NaCN and KCN, even in small amounts, by accurately detecting even the most minute changes in refractive index (RI). The sensor's rapid reaction allows for immediate monitoring of KCN and NaCN levels, which is crucial for promptly dealing with hazardous circumstances. To summarize, the recently launched PCF detectors are very adaptable devices that provide excellent accuracy and effectiveness in precisely detecting KCN and NaCN in diverse scenarios. This greatly enhances the process of monitoring. In order to enhance the reliability, practicality, and accuracy of a PCF detector for the identification of noxious gases, forthcoming studies will explore novel materials, revamp the sensor's design, and employ sophisticated signal processing methodologies. We highly recommend our recommended sensor due to its ability to detect NaCN and KCN, as well as its adherence to fabrication methods.

## Funding

No pertinent.

## Ethics approval

No pertinent.

## Ethics statement

No pertinent.

## Availability of data

The relevant author can be contacted to obtain the data supporting the results of this work. Owing to [state limitations like privacy or ethical limits], the data are not publicly available.

## Code availability

No pertinent.

## CRediT authorship contribution statement

**Md Safiul Islam:** Writing – original draft, Software, Methodology, Investigation, Data curation. **A.H.M. Iftekharul Ferdous:** Writing – original draft, Validation, Supervision, Project administration, Methodology, Conceptualization. **Khalid Sifulla Noor:** Writing – review & editing, Software, Resources, Formal analysis. **Most Momtahina Bani:** Writing – review & editing, Software, Resources, Formal analysis.

## Declaration of competing interest

The authors declare that they have no known competing financial interests or personal relationships that could have appeared to influence the work reported in this paper.
